# Testing a Machine Learning–Based Adaptive Motivational System for Socioeconomically Disadvantaged Smokers (Adapt2Quit): Protocol for a Randomized Controlled Trial

**DOI:** 10.2196/63693

**Published:** 2025-04-16

**Authors:** Ariana Kamberi, Benjamin Weitz, Julie Flahive, Kavitha Balakrishnan, Julianna Eve, Reem Najjar, Tara Liaghat, Daniel Ford, Peter Lindenauer, Sharina Person, Thomas K Houston, Megan E Gauvey-Kern, Jackie Lobien, Rajani S Sadasivam

**Affiliations:** 1 Division of Health Informatics and Implementation Science Department of Population and Quantitative Health Sciences UMass Chan Medical School Worcester, MA United States; 2 Division of Biostatistics and Health Services Research Department of Population and Quantitative Health Sciences UMass Chan Medical School Worcester, MA United States; 3 Department of Healthcare Delivery and Population Sciences University of Massachusetts Chan Medical School-Baystate Springfield, MA United States; 4 Institute for Clinical and Translational Research School of Medicine Johns Hopkins University Baltimore, MD United States; 5 Department of Internal Medicine Wake Forest University Winston-Salem, NC United States

**Keywords:** smoking cessation, mHealth, socioeconomically disadvantaged, biochemical verification, machine learning

## Abstract

**Background:**

Individuals who are socioeconomically disadvantaged have high smoking rates and face barriers to participating in smoking cessation interventions. Computer-tailored health communication, which is focused on finding the most relevant messages for an individual, has been shown to promote behavior change. We developed a machine learning approach (the Adapt2Quit recommender system), and our pilot work demonstrated the potential to increase message relevance and smoking cessation effectiveness among individuals who are socioeconomically disadvantaged.

**Objective:**

This study protocol describes our randomized controlled trial to test whether the Adapt2Quit recommender system will increase smoking cessation among individuals from socioeconomically disadvantaged backgrounds who smoke.

**Methods:**

Individuals from socioeconomically disadvantaged backgrounds who smoke were identified based on insurance tied to low income or from clinical settings (eg, community health centers) that provide care for low-income patients. They received text messages from the Adapt2Quit recommender system for 6 months. Participants received daily text messages for the first 30 days and every 14 days until the end of the study. Intervention participants also received biweekly texting facilitation messages, that is, text messages asking participants to respond (yes or no) if they were interested in being referred to the quitline. Interested participants were then actively referred to the quitline by study staff. Intervention participants also received biweekly text messages assessing their current smoking status. Control participants did not receive the recommender messages but received the biweekly texting facilitation and smoking status assessment messages. Our primary outcome is the 7-day point-prevalence smoking cessation at 6 months, verified by carbon monoxide testing. We will use an inverse probability weighting approach to test our primary outcome. This involves using a logistic regression model to predict nonmissingness, calculating the inverse probability of nonmissingness, and using it as a weight in a logistic regression model to compare cessation rates between the two groups.

**Results:**

The Adapt2Quit study was funded in April 2020 and is still ongoing. We have completed the recruitment of individuals (N=757 participants). The 6-month follow-up of all participants was completed in November 2024. The sample consists of 64% (486/757) female participants, 35% (265/757) Black or African American individuals, 51.1% (387/757) White individuals, and 16% (121/757) Hispanic or Latino individuals. In total, 52.6% (398/757) of participants reported having a high school education or being a high school graduate; 70% (529/757) smoked their first cigarette within 30 minutes of waking, and half (379/757, 50%) had stopped smoking for at least one day in the past year. Moreover, 16.6% (126/757) had called the quitline before study participation.

**Conclusions:**

We have recruited a diverse sample of individuals who are socioeconomically disadvantaged and designed a rigorous protocol to evaluate the Adapt2Quit recommender system. Future papers will present our main analysis of the trial.

**Trial Registration:**

ClinicalTrials.gov NCT04720625; https://clinicaltrials.gov/study/NCT04720625

**International Registered Report Identifier (IRRID):**

DERR1-10.2196/63693

## Introduction

### Background

Cigarette smoking is a serious public health problem and the leading cause of preventable disease, disability, and death in the United States [1]. While overall smoking rates have decreased, smoking rates among individuals who are socioeconomically disadvantaged remain high [2-5]. Individuals who are socioeconomically disadvantaged are affected disproportionately from smoking-related diseases [2-5]. Individuals who are socioeconomically disadvantaged may be less likely to try to quit smoking (eg, due to lower self-efficacy and maladaptive beliefs) and are less likely to use evidence-based strategies (such as quitlines).

### Computer-Tailored Health Communication via Texting: A Potential Approach

One strategy to engage these individuals is by using computer-tailored messages delivered via texting. Computer-tailored health communication involves the use of computer programs to select the best personalized message for an individual. This form of communication has been effective in enhancing motivation to quit and supporting cessation [6-12]. In a systematic review, computer-tailored messages showed higher cessation rates than nontailored messages (relative risk 1.17, 95% CI 0.97-1.41). Computer-tailored messages work because participants are more likely to engage and carefully process personally relevant messages [13]. Thus, methods to increase the relevance of the message may increase the effectiveness of the smoking cessation intervention. Computer-tailored messages delivered via texting over phones may be particularly attractive for individuals who are socioeconomically disadvantaged. Mobile phone ownership is now nearly universal (95%) [14], and these rates are consistent across socioeconomically disadvantaged groups (less than high school education, 92% and those earning <US $30,000, 92%). The texting app is the most commonly used app on mobile phones [14].

### The Adapt2Quit Intervention: Leveraging Machine Learning for Tailored Cessation Support

In our National Institutes of Health–funded trial, we are testing Adapt2Quit, which uses machine learning algorithms (ie, recommender systems) to computer-tailor messages for an individual and deliver them via texting [15]. Recommender systems can be programmed to continuously learn from user feedback and improve personal relevance and customer engagement [15]. Adapt2Quit also includes proactive texting facilitation to the state quitline in which participants are asked if they want to be referred to the quitline, and those who agree are referred to the quitline. Quitlines are programs in the United States that offer evidence-based support (eg, counseling and medications) to individuals who smoke. These individuals can either directly contact quitlines using the number 1-800-QUIT-NOW or be referred by a health care provider or tobacco cessation programs. Although quitlines have been established in all 50 US states, they are vastly underused. The use of texting to promote quitline use could potentially increase quitline use, and thereby increase the effectiveness of the smoking cessation intervention for the participants.

### Preliminary Evidence Supporting Adapt2Quit

In our preliminary studies, Adapt2Quit increased engagement and 30-day point-prevalence effectiveness over a standard messaging that randomly selected these messages [16]. In a subanalysis among those who reported lower education (n=49), the average rating of the messages was higher on more days than the average ratings of true comparison messages (77% vs 23%; *P*<.01). This pilot study showed the potential of the Adapt2Quit trial among individuals who are socioeconomically disadvantaged. Since the beginning of the trial, we have completed another randomized controlled trial that tested the machine learning algorithm compared to standard messaging among those who were recruited via social media [17]. In the other trial, there was no significant difference in 6-month point-prevalence smoking cessation (adjusted odds ratio 0.81, 95% CI 0.61-1.08) between the recommender system (146/412, 35.4% of participants with outcome data) and messages tailored by standard messaging based on participants’ baseline readiness to quit groups (156/389, 40.1% of participants with outcome data). However, the other trial differs from the current trial being reported in that the messages were delivered via email, it did not target individuals who are socioeconomically disadvantaged, and the system did not facilitate referral to the quitline. Furthermore, another group has evaluated a similar recommender system approach for message selection in a pilot trial for self-reported smoking cessation [18]. However, additional studies testing recommender systems are needed to uncover the potential of these systems for increasing the effectiveness of computer-tailored smoking cessation interventions.

### Theoretical Basis for the Adapt2Quit Intervention

The Adapt2Quit intervention is based on the self-determination theory (SDT). SDT-based interventions support autonomous decisions and are designed to increase intrinsic motivation and self-regulation and they have been shown to improve motivation and cessation outcomes among those who smoke [19-24]. Three innate psychological needs are theorized to promote self-motivation: autonomy, relatedness, and competence [19]. Autonomy reflects the need to engage in behaviors with a sense of choice or personal endorsement [25]. Autonomy support can be provided by acknowledging an individual’s unique perspective and offering choice [25,26]. Relatedness reflects the degree to which an individual feels connected to and understood by others. Competence is a feeling of being capable and is akin to self-efficacy, the belief that one can bring about desired outcomes. By supporting autonomy, relatedness, and competence, health behaviors are more likely to be internalized and maintained. Specific examples of how Adapt2Quit supports the SDT are provided in Textboxes 1 [27] and 2. Our aims and hypotheses align with our proposed model, which suggests that relevant personal messages from the Adapt2Quit system will enhance system engagement. This increased engagement is expected to improve internal processes (ie, the SDT constructs measured using the Perceived Competence Scale [PCS]), leading participants to adopt cessation-supporting behaviors (external processes such as quitline and nicotine replacement therapy [NRT] use), ultimately resulting in smoking cessation.

**Examples of mapping the self-determination theory to Adapt2Quit.**

- Autonomy: participants can control how often they want to rate the messages with the system. Autonomy increases as the participant’s ratings influence the messages they receive, providing an enhanced feeling of control over the intervention. Furthermore, Adapt2Quit facilitates quitline access but does not impose it. Participants are asked if they want to contact the quitline. Only if the participant responds positively, our system will initiate a quitline referral. Autonomy is increased as the participant controls if and when they choose to interact with the quitline. Our messages will focus on persuading the smoker to use quitline.
- Relatedness: in addition to the smoker profile, Adapt2Quit’s recommendations are based on feedback from thousands of smokers who previously engaged with the recommender system. Relatedness increases as the system learns and adapts to the ratings feedback of each new smoker. The Adapt2Quit messages database includes peer-written messages [27]. Messages from peers may enhance relatedness because they reflect shared experiences, allowing smokers to more easily identify with the message content [27].
- Competence: Adapt2Quit messages are designed to improve motivation, educate, and increase a smoker’s confidence and skills related to smoking cessation. Content includes appropriate strategies and treatments, as cues and reminders to encourage use, which were developed based on current guidelines on smoking cessation. Our pilot showed that Adapt2Quit increased the adoption of cessation-supportive behavior.

**Specific aims.**

**Aim 1: Adapt2Quit engagement—heterogeneity of Adapt2Quit recommender system engagement**

- Hypothesis 1a: among Adapt2Quit participants, those with higher engagement levels (ie, completed more ratings) will have greater scores on the Perceived Competence Scale (PCS).

**Aim 2: behavioral processes—internal processes (self-determination theory constructs) and external “processes” or cessation-supportive actions comparing Adapt2Quit and the comparison group (texting facilitation to quitline)**

- Hypothesis 2a: Adapt2Quit participants will have greater scores on the PCS than comparison participants.
- Hypothesis 2b: Adapt2Quit participants will adopt more cessation-supporting actions (quitline use and nicotine replacement therapy) than comparison smokers.

**Aim 3: effectiveness—6-month smoking cessation in Adapt2Quit versus the comparison group**

- Hypothesis 3a: (primary outcome) Adapt2Quit participants will have greater smoking cessation rates (6-month point-prevalence biochemically verified) than comparison participants.
- Hypothesis 3b: (mediation analysis) measured internal and external processes will mediate the effect of Adapt2Quit on smoking cessation.

### Paper Objective

This paper describes the protocol and status of our ongoing randomized controlled trial testing the Adapt2Quit intervention among individuals from socioeconomically disadvantaged backgrounds who smoke. Our comparison participants receive the biweekly texting facilitation messages but do not receive the motivational messages recommended by the Adapt2Quit intervention. Our protocol description includes the intervention and comparison system as well as the description of the analyses of the primary and secondary hypotheses listed in the following sections. Since recruitment for our study has finished, we describe the demographics of the participants we have recruited. Our follow-up data collection is in progress.

## Methods

### Study Design and Setting

This study is a 2-arm randomized controlled trial comparing participants who received smoking cessation text messages selected by the Adapt2Quit recommender system plus a texting facilitation approach to connect participants to the quitline with participants who received the texting facilitation but no smoking cessation text messages. Study participants were blinded to group assignments. They were recruited from health systems in diverse geographic areas. Recruitment started in April 2020 and was completed in March 2024. Our target sample size was 750 individuals from socioeconomically disadvantaged backgrounds who smoke.

### Ethical Considerations

The Adapt2Quit study has been approved by all participating institutions’ institutional review boards (IRBs), including Baystate (BH-20-123) and Johns Hopkins (IRB00280942), with the University of Massachusetts Chan Medical School (UMass Chan) IRB (H00018991) being the central IRB. The study is listed in ClinicalTrials.gov (NCT04720625). It is being carried out in accordance with the Helsinki Declaration, and all participants have signed an informed consent form before enrollment in the study. Participants have been informed that they have the right to withdraw their consent at any point during the study, and the reason for their withdrawal will also be documented if the participant so wishes. The participants have received compensation for completing study activities. They received US $25 for completion of baseline, US $25 for the 6-month follow-up, US $50 for completing the carbon monoxide (CO) verification assessment, and an additional US $50 for participation in the qualitative interview. Upon randomization, all patients have been assigned an individual ID number so their data remain anonymous.

### Inclusion and Exclusion Criteria

The inclusion criteria were as follows: an individual who smokes, comes from a socioeconomically disadvantaged background, speaks English, who is active in care (having at least 2 clinical visits in the past 2 years), and who has a texting-enabled mobile l phone. We used insurance status to identify participants as socioeconomically disadvantaged [28]. In the United States, people can receive health insurance or health care payment coverage from several sources including through their employer, other private health insurance companies, or through government-supported programs. Individuals who have these government-supported programs are more likely to be from socioeconomically disadvantaged groups. For our study, we used Medicare (a government-funded program for older adults and those with disabilities) and Medicaid (a state and federal program for low-income individuals) to identify individuals from socioeconomically disadvantaged backgrounds. We determined smoking status by asking participants if they had smoked at least 100 cigarettes and their current smoking frequency (Multimedia Appendix 1). Those who responded “yes” to 100 cigarettes and “some” or “every day” were included.

The exclusion criteria were as follows: adults who were unable to consent, individuals who were not yet adults (ie, aged <18 years), prisoners, people with Alzheimer disease, and pilot study participants.

### Recruitment

Recruitment was spread over the 3.5 years of the study. Study participants were recruited at urban and rural health care settings across 3 health care systems in the United States (UMass Memorial Medical Center, Baystate Health, and Johns Hopkins University). We used an opt-out recruitment strategy, with some variations between the three sites. At all 3 sites, after obtaining a Health Insurance Portability and Accountability Act waiver, potential participants who met our inclusion criteria in the clinical data warehouse were identified. At the UMass Chan and Baystate Medical Center, an initial invitation letter with a self-addressed, stamped opt-out postcard was sent to those eligible from the local site principal investigator outlining the study’s purpose. Johns Hopkins University’s site used emailed study invitation letters with instructions to opt-out via email instead of or in addition to mailed letters and “opt-out” postcards for those without an email address. Instructions were provided on how to opt-out by email. At all sites, individuals who opted out or whose email was returned undelivered within 2 weeks were removed from the recruitment list.

### Screening, Informed Consent, and Baseline Data Collection

Potential participants were phoned up to 4 times to determine their interest and were screened for eligibility. Before starting the first recruitment call, the research staff sent an automated introductory text message to potential participants who would be called that day. During the phone call, participant eligibility was assessed (Multimedia Appendix 1). Eligible participants were asked to provide informed consent for study participation, were asked to complete the baseline data, and were then randomized to treatment. The screening session, informed consent, and baseline data collection were conducted via Zoom (Zoom Communications, Inc) or by phone.

Once randomized, after completion of the baseline survey (Multimedia Appendix 1), the Adapt2Quit intervention participants received 5 initial motivation messages and were asked to provide a rating. The recruitment staff viewed the rating in real time to confirm whether the participant understood and was comfortable with the system. The staff emphasized the importance of rating their messages to intervention participants. The research staff explained the quitline facilitation process and smoking status texting assessment to both groups and reiterated the importance of completing the ratings to intervention participants. Those randomized to the control group received only the texting facilitation and smoking status but did not receive the motivational messages, and instructions were provided accordingly.

### Randomization

Separate randomization tables were created for each site to enhance the balance between the two groups. Participant allocation to study arms was based on a permuted block scheme in which treatment assignments were made within blocks so that numbers assigned to each treatment arm are equal after a block has been filled. Blocks of various sizes (2, 4, and 6) were used in random order to facilitate allocation concealment. As in our prior technology-based trials, we embedded randomization within the technology [29,30]. After completing the screening, informed consent, and baseline data collection, the research staff entered the participant ID and participant mobile phone number from the survey into a web-based system, which then assigned the allocation based on the randomization table. Using this technique, participants and staff were blinded to allocation during the initial session. The research staff were subsequently unblinded to provide personalized training for the intervention and control groups, as noted.

### Intervention and Comparison

#### Intervention: The Adapt2Quit Recommender System

The Adapt2Quit recommender system uses a machine learning algorithm to select the best message for a participant from a motivational messaging database. We programmed Adapt2Quit to select messages based on the following: (1) the smoking status of the participant and (2) the ratings database (both from prior study participants and current participants) [16,31-33]. We discuss the recommender system components in subsequent sections. The system has multiple components.

#### Component 1: The Motivational Messages Database

The messages include 261 messages, including 206 expert- and 55 peer-written messages (Multimedia Appendix 2) [27]. We developed the expert-written messages through an iterative group review process guided by theoretical frameworks and existing smoking cessation guidelines [34]. Peer-written messages were written by current and former people who smoke, responding to a web-based survey. Our methods for developing this messaging database and its characteristics have been published [27]. Briefly, the expert messages written by smoking cessation providers and researchers were more “biomedical” (ie, avoidance, behavioral strategies, and health), while peer messages had more “social” and “real-life” content (ie, expectations, money, quality of life, attitudes, and friends) [27]. Our messages were assessed to be readable for someone with at least a fifth grade–level education (Flesch-Kincaid Grade level 5.5). In total, 19.9% (52/261) of the messages were peer messages. Overall, 52.9% (138/261) of the messages provided information about behavioral treatments (eg, substitution and distraction); 39.8% (104/261) contained motivational content, such as reasons to quit; 26.8% (70/261) included health-related information, such as the risks of smoking to physical and cognitive functioning; and 19.9% (52/261) addressed how seeking social support or smoking impacted social interactions with family and friends. Multimedia Appendix 2 lists example messages.

#### Component 2: Ratings Database

The algorithm uses 2 types of ratings data to generate the following recommendations.

First, ratings from prior study participants. More than 1000 participants have rated our messages using the influence rating question (see definition in the Data Elements, Statistical Analysis, and Power Calculation for Specific Aims section) resulting in 18,920 ratings [16,32,33]. We chose the influence rating question after a pilot test in which we asked participants to rate each message on 4 aspects: influence, emotional response, relevance, and preference [33]. We found that all these aspects were highly correlated. In developing the algorithms for the recommender system, we compared ratings by different user profile characteristics. Of the 20 messages rated most highly by participants with less than a high school education, only 3 messages were rated in the top 20 by college graduate participants. Adapt2Quit considers all these variations for message selection.

Second, current ratings from participants in the intervention group using 2-way texting. Adapt2Quit is also programmed to learn from the explicit ratings of participants receiving the messages. Thus, when a participant is sent a text message, it will be accompanied by a question (see definition in the Data Elements, Statistical Analysis, and Power Calculation for Specific Aims section) asking the participant to rate the text message using the influence ratings question. The participant can choose how frequently they wish to rate the text messages. In addition to improving the system, our participants in the pilot studies noted that rating the messages helped them to cognitively process the messages better and remember their content.

#### Component 3: Machine Learning Algorithm

In our prior publications, we have described our algorithm development in detail [16,32,33]. For the purpose of this paper, we describe our approach and the functioning of the system in brief. We used a strong generalization protocol that involved completely separating test users from train users, learning a model using all the train users’ ratings, freezing all non–user-specific parameters, and finally training the user-specific parameters on a subset of each test user’s observed ratings. We evaluated several recommender methods for accurate prediction, including K-nearest neighbors, probabilistic matrix factorization, collective matrix factorization, and the Bayesian probabilistic matrix factorization (BPMF) [33]. In evaluating rating prediction methods, we used a range of standard performance metrics including root mean squared error, Kendall tau-b, and normalized discounted cumulative gain. In all these tests, BPMF was identified as the best single model. For example, comparing the root mean squared error metric between the different algorithms, there was a small but statistically significant gap (*P*=.01) between the BPMF and other algorithms as determined by a paired *t* test with Bonferroni correction. The BPMF model estimates a probability distribution over a joint embedding of users and items into complementary latent spaces. The rating of the given user supplies for a given item is approximated by the expected value of the product of the latent user and item factor vectors representing the user-item pair, with the expectation taken over the uncertainty in embeddings.

The intervention participant experience is as follows: during baseline registration, the intervention participant will be asked to rate 5 messages. The recommender system will use these ratings to select the first message. As the intervention unfolds, the participant will be asked to rate each message, and the system will learn and adapt to these ratings [16,32,33]. In the backend, each time the program needs to select a message for an individual, the program sends all the prior ratings provided by the participant and the messages rated by the participant. The system also sends demographic information of the participant. The algorithm then selects the next messages based on these data from the list of messages that have not been sent to the participant. The algorithm was also programmed to choose only from among those messages that matched the participant’s readiness to quit status. All participants will receive a new message every time. When they do not rate the message on a particular day, our system will have the 5 messages rated at baseline and the ratings of the prior participants to fall back upon to pick a new message. Because the machine learning algorithms operate as a black box, in our prior study, we conducted an analysis of our messages to uncover the black box of the algorithms and understand the message selection process and have published our results [35].

#### Flow of the Intervention

Participants in the intervention group receive motivational messages, texting quitline facilitation, and texting assessment of smoking status.

Motivational messages selected by the Adapt2Quit recommender system including ratings for user feedback. Participants receive daily motivational messages for the first 30 days and every 14 days until the end of the study. As noted, participants were asked to read these messages and provide their ratings. They were able to choose how frequently they rated messages.

For texting quitline facilitation, participants in both study arms were asked (via text message) if they were interested in being referred to the quitline every 14 days or until the participant responded “yes” to this question. We have followed current best practices in SMS text messaging to connect users to the quitline as described in the study by Krebs et al [36], who, after assessing multiple message frames, found that including a self-efficacy frame was the most effective. If participants request to be connected, a member of the study team refers them to quitline via a secure web form provided by the Massachusetts and Maryland quitline. As per their standard protocol, once quitline staff receive notice of participant interest, they proactively call the participant and engage in counseling.

For texting assessments of smoking status, participants were asked, “What is your current smoking status?” every 30 days over a 6-month period.

### Comparison

To isolate the effect of the Adapt2Quit recommender system, comparison participants do not receive tailored motivational messages but receive the texting quitline facilitation and the texting assessment of smoking status with the same frequency as intervention participants.

### Six-Month Data Collection Procedures

#### Overview

Six-month follow-up was conducted via telephone using the REDCap (Research Electronic Data Capture; Vanderbilt University) system to collect the data. Participants had the option to complete the REDCap survey on their own, with research staff sending the secure REDCap link by either email or text or mailing a paper survey to the participant. The system is designed to first collect the main outcome data and then branch into any intervention or control-specific questions. This way the staff is blinded to the participants’ assignment when the primary outcome data are collected. To maximize retention, participants were asked upon enrollment to provide 2 alternate telephone numbers, their physical and email addresses, and any other contact information, as applicable. All this contact information was used in making multiple attempts to reach participants to complete their follow-up assessment, which includes sending a thank you letter and incentives for completing the 6-month visit. Participants in both groups will receive a US $25 incentive for completion of the 6-month follow-up and an additional US $50 for completing the CO verification assessment. A web-based tracking system was used to automatically alert staff about participants due for the 6-month follow-up measure to facilitate timely assessments.

#### Qualitative Interviews

After completion of the 6-month follow-up visit, intervention participants will be invited to complete the qualitative interview to deepen the understanding of potential mechanisms and ways to improve the next generation of the Adapt2Quit intervention (Multimedia Appendix 1). We are using a purposeful sampling approach to recruit interview participants (n=30). Purposeful sampling is a technique widely used in qualitative research for the identification and selection of information-rich cases for the most effective use of limited study resources [37]. We will select participants based on the extent of engagement with Adapt2Quit, use of the quitline, and eventual quitting success. Study coordinators will contact participants who agree to be interviewed to complete the audio-recorded qualitative interviews. The interviews will be conducted by phone and will examine a series of topics following the hypothesized path model presented in the research strategy (Adapt2Quit increases engagement, which results in external and internal processes causing the participant to implement smoking cessation–supporting behaviors; Multimedia Appendix 1). Across these topics, our goal is to understand what participants value most and to elicit recommendations for further system enhancement. Interviews are semistructured, allowing for natural conversation with opportunities to explore unanticipated issues, and will be no longer than 30 to 45 minutes in duration. All qualitative interview participants will be asked to provide verbal consent before participating in the interview and being audio recorded. For participation in the qualitative interview, participants will receive US $50.

### Data Elements, Statistical Analysis, and Power Calculation for Specific Aims

Data are collected throughout the study at several time points via text-based ratings, quitline use, assessments, questionnaires, and CO readings. Our main outcomes are described subsequently.

#### Completion of Ratings

Each Adapt2Quit message is accompanied by the influence ratings question as follows: “Please type the number below to indicate whether you agree with this statement—How much does this message influence you to quit smoking”—on a 5-point Likert scale (strongly disagree to strongly agree). Our program tracks the ratings completed for each participant.

#### Internal Processes

Perceived competence, a key SDT construct, will be measured using the previously validated PCS [23]. The 4-item scale assesses the participant’s feelings of being able to stop smoking permanently (“I feel confident in my ability to not smoke,” “I now feel capable of not smoking,” “I am able to not smoke anymore,” and “I am able to meet the challenge of not smoking”). Responses are scored on a 7-point scale from 1 (strongly disagree) to 7 (strongly agree), and individual items are averaged to create a scale mean. In prior work [23], PCS was reliable (α=.95) at the 6-month follow-up [23]. We will assess PCS at baseline and at the 6-month follow-up visit.

#### External Processes (Quitline and NRT)

Quitline use is assessed using data collected from the quitline. We will send a list of our users and ask quitline to provide data on these users, such as the number of contacts between the smoker and the counselor. We will use the time to first call completed with the quitline counselor as our primary quitline measure. NRT is self-reported (yes or no).

#### Six-Month Cessation

Assessment of the 6-month smoking cessation is collected using the 7-day point-prevalence question [38]: “Do you currently smoke cigarettes (smoked even 1 puff in the last 7 days)?” Point prevalence can capture an intervention’s delayed effects. A psychometric analysis comparing continuous, prolonged, and point-prevalence outcomes found that point prevalence had the highest concurrent validity [38]. The 7-day window provides an appropriately stringent measure to account for a cross-sectional snapshot. Using the CoVita Smokerlyzer breath CO monitor, participants who self-report quitting at the 6-month follow-up will be asked to complete biochemical verification remotely (the device will be mailed to them) or in person. Because of the variability of the device, participants will be asked to repeat the test 3 times. The average of these values will be used for our analyses [39]. Participants will be classified as tobacco users if their CO measurement is >6 ppm [40]. Participants completing remotely will be asked to conduct the test over videoconference, where our staff will monitor and guide them. To complete the biochemical verification, whether in person or remotely, study participants will receive a US $50 gift card.

### Data Analysis

#### Statistical Analysis and Power Calculations

To preserve randomization, all primary analyses will be on an intent-to-treat basis. Secondary analyses will explore dose-response effects among those with variable levels of adherence to the intervention. All analyses will be 2-sided, and the α error level will be set at .05. We will begin by examining univariate statistics (means, medians, SDs, frequencies, percentages, and 95% CIs) and distributions of the variable of interest. We will examine the balance of participant characteristics by study groups and account for any imbalances in our multivariable analysis. To address sex as a biological variable, we will stratify the analyses by male and female to evaluate whether the mechanisms and outcomes are modified by sex. We will test group differences either using chi-square tests of independence (categorical variables), *Z* test, or a 2-tailed *t* test (continuous variables), or the equivalent nonparametric tests depending on the distribution of the variables. Baseline differences between the intervention and control group will be established based on standardized differences, rather than on tests of statistical significance.

#### Aim 1: Adapt2Quit Engagement

Aim 1 is analyzed within the intervention group (N=375). Engagement will be defined as the number of daily ratings divided by the total number of rating opportunities (percentage of ratings completed).

##### Hypothesis 1a

Among Adapt2Quit participants, those with higher engagement levels (completed more ratings) will have greater scores on the PCS. For hypothesis 1a, we will explore the distribution of the PCS at baseline and the averaged PCS scores during follow-up. Furthermore, we will analyze the distribution of engagement. It is likely that these distributions will be nonnormally distributed. In this case, we will transform the distribution into something approximating normality or choose a nonparametric test to assess the correlation between engagement and the PCS. We will generate a generalized linear model with the averaged follow-up PCS score as the dependent variable and engagement as the independent variable, adjusting for demographic characteristics that vary between highly engaged and less-engaged individuals and for baseline PCS measurements.

##### Power for Hypothesis 1a

We have considerable *power* to detect relatively small correlations for secondary analyses. As a preliminary power estimate, if we assume a normal distribution and α=.05, we have 80% power to detect a correlation coefficient as small as 0.2 with 200 patients. For larger, more meaningful correlations, we have well over 90% power starting at a sample of 375.

#### Aim 2: Behavioral Processes

##### Hypothesis 2a

Adapt2Quit participants will have higher scores on the PCS than control participants. To analyze hypothesis 2a, we will first create a generalized linear model where the dependent variable is the averaged PCS score during follow-up (see the section Hypothesis 1a), the independent variable is randomization group, and the baseline PCS score is included as a covariate. Furthermore, to fully use the repeated measures of the PCS, we will develop a random effects interrupted time series model evaluating the change in slope of PCS over time, comparing the Adapt2Quit intervention and comparison group. This will be implemented using a segmented regression to evaluate changes in slope early in the intervention and sustain change over time in the latter months of the 6-month follow-up.

##### Power for Hypothesis 2a

In a prior smoking cessation study, the mean (SD) of PCS was 4.79 (1.96) [23]. On the basis of these results, for power calculations, we assumed a meaningful difference in the averaged PCS score to be equal to 1 SD, assumed the mean in control to be 4.79, and set α at .05. To detect a difference of 1 SD, we have 99% power with a sample of 120 participants per group, further demonstrating power for aims 1 and 2.

##### Hypothesis 2b

Adapt2Quit participants will adopt more cessation-supporting actions (quitline and NRT) than control participants. For hypothesis 2b, the independent variable is again the randomization group. Each dependent variable will be modeled separately. There are several ways to analyze quitline use, including time to first call completed (time from baseline when the first call with the counselor was conducted in a number of weeks) or whether the participant used the quitline and the number of contacts between the patient and counselor. We chose the time to first call completed with the quitline counselor because it matters how quickly the intervention succeeded in motivating participants to call the quitline (if the call happened in the first few days of the intervention or later). The participant may initiate the call, or the counselor can call the participant following a texting referral.

##### Power for Hypothesis 2b

Analysis and the power calculation for time to first call completed is similar to the hypothesis 3b analysis. The use of NRT will be assessed via self-report at the 6-month follow-up. To analyze NRT use, a dichotomous variable, we will create a logistic model with the use of NRT (yes or no) as the dependent variable and randomization as the independent variable.

#### Aim 3: Effectiveness

##### Hypothesis 3a: Primary Outcome

Adapt2Quit participants will have greater smoking cessation rates (6-month point-prevalence biochemically verified) than control participants. For our primary outcome, we will follow recommendations from the Society for Nicotine and Tobacco Research and other experts. While we will attempt to minimize dropout rates loss to follow-up (missing outcomes) in trials such as ours cannot be fully averted [41]. Penalized imputation (missing=still smoking) is not a conservative approach and can be biased (against a group), especially when there is a differential missing between the groups [41]. Thus, the recommended approach is to use inverse probability weighting [42]. In this approach, the first step is to develop a logistic regression model to predict nonmissingness using variables that are found to be different between study participants with and without missing data. The next step is to calculate the inverse probability of nonmissingness and use it as the weight in a logistic regression model to compare the cessation rate between two study groups [43]. Along with inverse probability weighting, we will present penalized imputation and a complete case analysis (limited to those with complete data) as sensitivity analyses. We propose to biochemically verify participants who self-report quitting smoking using a CO meter.

##### Power for Primary Outcome (Hypothesis 3a)

In our prior National Cancer Institute trial, we observed an absolute 9% difference in the standard messaging versus no-messaging control group [29]. We expect socioeconomically disadvantaged groups to have lower cessation rates than non–socioeconomically disadvantaged groups. Thus, we calculated power using a range of control cessation rates from 7% to 9%. We started at 7%, because it is the estimated rate at which the general population of all individuals who smoke quit without any intervention [34]. On the basis of the recommender enhancements (and further pilot data), we saw an additional marginal difference of 6% at 30 days (even higher for African American individuals who smoke) and expect this difference to continue and expand over time as the recommender system continues to learn and adapt to the individual; however, to be conservative and recognizing the potential difficulty in impacting behavior in this population, we chose to power for a small difference, namely 7%. Although our hypothesis is directional, we have chosen to adopt the commonly used conservative approach of 2-sided tests for all our power calculations. On the basis of a 2-sided chi-square test of equal proportions, α=.05, for all these base rates, we will have 80% power with 600 participants (N=300 per arm). Considering loss to follow-up, we plan to oversample by 25% and thus have budgeted for a total of 750 participants to complete baseline and be randomized. On the basis of our prior experience recruiting from clinical systems, we expect the dropout rate to be lower than the 25% we have assumed in our calculations.

##### Hypothesis 3b: Mediation Analysis

**Figure 1:**
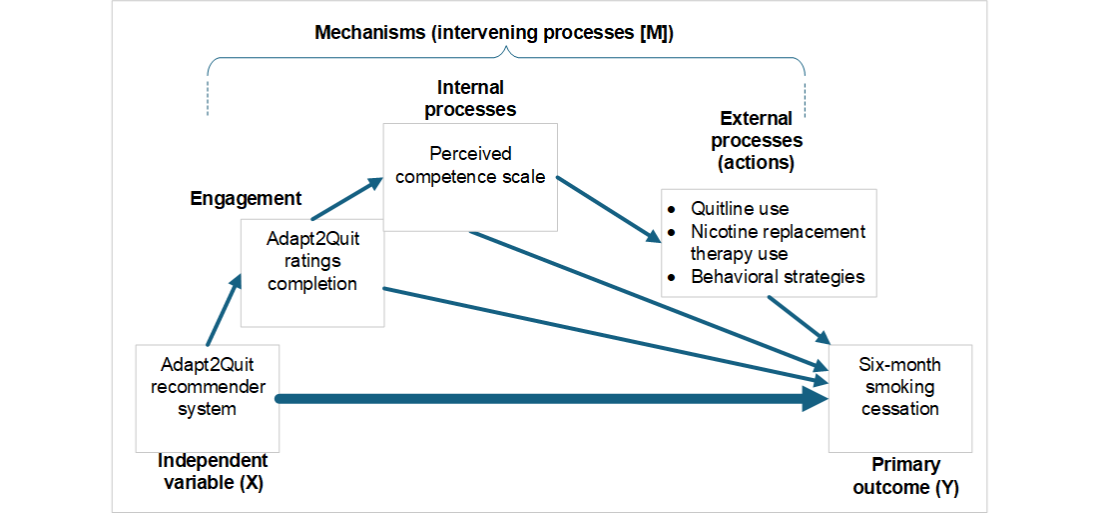
Hypothesized path model depicting associations among constructs. NRT: nicotine replacement therapy.

Measured internal and external processes will mediate the effect of Adapt2Quit on smoking cessation. Adapt2Quit is expected to result in several interconnected processes (Adapt2Quit increases personal relevance, thereby improving system engagement, which results in increasing the external and internal processes, causing the participant to implement cessation-supporting behaviors resulting in smoking cessation; Figure 1). We will explore the pathways using modern mediation and structural equation modeling (SEM) techniques. Mediation occurs when an independent variable (X) leads to a given outcome (Y) through an intervening process (M). In our case, the independent variable is the Adapt2Quit recommender (X), and the outcome will be 6 months of smoking cessation (Y). Perceived competence and adoption of cessation-facilitating behaviors (use of quitlines, use of NRT, and adoption of behavioral strategies) are all potential mediators (M). The mediation analyses will focus on each individual process variable, using the classic mediation principles modified from Barron and Kenny [44]. This approach requires 3 regression models and decomposes the total effect (*c*) of the independent variable (X) into a direct component (*c’*) and an indirect or mediated component (c-c’). Limitations to the modified Barron and Kenny approach include the fact that more complex mediation pathways with multiple steps are not allowed. Thus, we will implement SEM using Mplus as the covariance analysis software. SEM allows for the simultaneous estimation of multiple regression equations representing complex mediation pathways, allowing errors to be correlated across equations. Furthermore, constructs may be represented as latent variables to relax the assumption of no measurement error. An array of diagnostic tests (eg, root mean square error of approximation and comparative fit index) are available to examine model quality.

#### Qualitative Interview Analysis

We will transcribe the interviews verbatim and will use the rapid qualitative analysis method to code the transcripts. The rapid qualitative method uses triangulation and iterative data analysis to quickly develop a preliminary understanding of a situation from the insider’s perspective [45-47]. In this method, primary topics or “domains” will be taken from the topics addressed in the interview guide to create summary templates. Two research team members will each code at least 10% of the interview transcripts, and coding checks will be completed to ensure interrater reliability. We will use the summary templates to create a matrix to analyze each domain’s depth and breadth of data. From these, we will identify study themes and subthemes using the matrix. The research team will collaboratively and iteratively review, discuss, and sort the data to refine the initial themes and subthemes and highlight the most salient quotes. Recordings collected during interviews will be deidentified using subject ID numbers. No identifiable information, such as a full name, will be collected in the recording. Identifiers will be collected separately and will be stored in an encrypted form. Recordings will be transcribed and deidentified. They will be stored in a secured UMass Chan drive specified for the study within the UMass Chan IS regulated environment, where only UMass Chan study staff will have access to them. Recordings, and all other data collected in this study, will be retained and destroyed in accordance with Standard Operating Procedure Human Research Protection-800. Transcripts of audio or video recordings as well as the audio or video recordings themselves will be fully destroyed 6 years from the conclusion of the study period or at the request of the individual to whom that data belongs.

## Results

**Figure 2:**
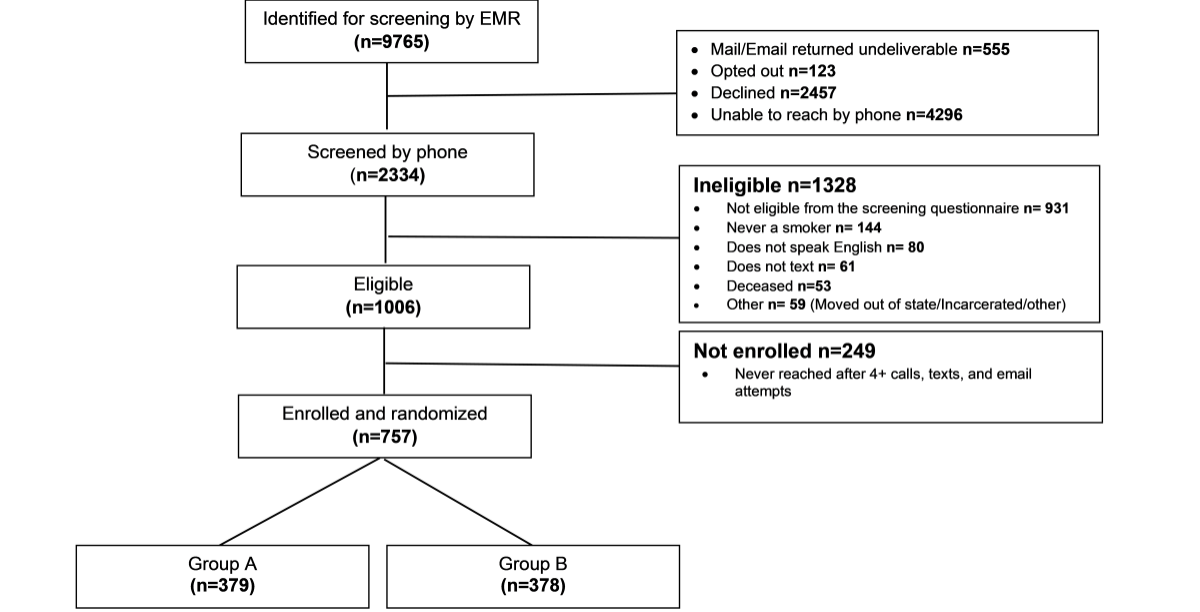
CONSORT (Consolidated Standards of Reporting Trials) diagram. EMR: electronic medical record.

This trial was funded in April 2020 and is currently ongoing. As of May 2024, we had enrolled 757 participants, and 591 (78.1%) of them had completed the 6-month follow-up interviews (Figure 2). We anticipate that data analysis and final manuscript preparation will occur in late 2025. Multimedia Appendix 3 provides the demographic and screening survey responses of 757 participants.

## Discussion

### Principal Findings

This study protocol describes a randomized controlled trial to test a machine learning algorithm–based, computer-tailored health communication system for smoking cessation. We hypothesized that the intervention will impact mechanisms of action—specifically engagement, competence (a theoretical construct), and adoption of cessation-supportive actions—as well as demonstrate effectiveness in promoting smoking cessation. Our system will test these hypotheses among individuals who are socioeconomically disadvantaged, a group disproportionately affected by smoking.

Because of the potential for wide reach and effectiveness, texting programs (especially texting) have been increasingly adopted in real-world settings worldwide (public health and within health care systems) [48-50]. For example, the World Health Organization has helped establish texting programs for smoking cessation in several countries, including Costa Rica, Tunisia, and India [51]. The US Department of Health and Human Services established a Text4Health Task Force in 2010 to promote health text messaging programs in the United States [48]. In 2017, 55% of state quitlines offered texting programs (up from 20% in 2012) [34,52]. There is now growing evidence that these programs can be effective. An evaluation of SmokefreeTXT (a free evidence-based smoking cessation program offered by the US National Cancer Institute) reported that point-prevalence abstinence for smokers who initiated treatment at completion of the 42-day program was 7.2% (3% at the 6-month follow-up) [53]. Leaders have called for continued innovations to increase the effectiveness of these health messaging programs, especially to increase effectiveness with smokers from socioeconomically disadvantaged backgrounds.

Our study will provide important evidence on whether a more personalized approach using machine learning can encourage quitline use and support smoking cessation, compared to a text-based referral to quitline. In previous papers, we described our pilot studies testing the Adapt2Quit intervention [16,35,54]. Furthermore, our prior large randomized controlled trial compared the Adapt2Quit intervention to a system that delivered messages based on participants’ baseline readiness to quit [17]. In that trial, we recruited participants online as well as through referrals from friends and family. In addition, messages were delivered by email rather than by text, as is being done in this trial. The previous study also did not include biochemical verification of smoking cessation. Beyond the potential benefit of computer-tailored messaging, our system aims to actively engage participants with the quitline. Quitlines have proven to be effective in supporting people to quit smoking, yet using a quitline and attending counseling sessions is a significant step for individuals from socioeconomically disadvantaged backgrounds who smoke. Currently, only 2% to 3% of people who smoke use the quitline, with usage rates even lower among individuals from socioeconomically disadvantaged backgrounds [34]. Prior research has shown that it is possible to use a low-intensity intervention such as texting to increase motivation and the use of cessation resources among people who smoke [55].

In addition to our system, one group compared a similar recommender system in a pilot trial to a system that selected messages based on a knowledge base. They compared the message appreciation, engagement with the system, and one’s own self-reported smoking cessation status (7-day point prevalence) [18]. Another group developed a recommender system for smoking cessation and promoting physical activity [56]. This group described an experiment they conducted to identify the optimal approach for selecting messages. In addition, a systematic review examined the potential of using another machine learning approach—reinforcement learning—for similar purposes [57]. Together, these approaches highlight the potential of the use of machine learning approaches to enhance a widely used behavioral intervention: computer-tailored health communication.

Because we have completed recruitment for our study, we also provide the characteristics of our participants. Our recruitment method, using an opt-out strategy at 3 different health care systems, was able to successfully recruit a diverse sample. A third of our sample (265/757, 35%) are Black, and 16% (121/757) self-identified as Hispanic or Latino. There may be several factors for our successes, including that our intervention is a low-intensity intervention using a texting model that is widely available. Furthermore, our procedures for screening, baseline, and follow-up are all remote, which may have reduced barriers to recruitment. Participants in our study indicated higher motivation levels than the general population. This is an artifact of our recruitment strategy that people who are more motivated participate in a study about tobacco cessation. This more representative sample implies that our study findings will be more generalizable to the US population.

### Strengths and Limitations

We describe the protocol for a rigorous evaluation of behavioral intervention among individuals from socioeconomically disadvantaged backgrounds. Since recruitment for our study is complete, we also present participant characteristics. Using an opt-out recruitment strategy across 3 different health care systems, we successfully recruited a diverse sample, which has been challenging to achieve for clinical trials. Our fully remote procedures for screening, baseline, and follow-up may have reduced barriers to recruitment. Notably, 35% (265/757) of our sample identified as Black and 16% (121/757) as Hispanic or Latino. This more representative sample suggests that our study findings will be more generalizable to the US population. One limitation is that our participants reported higher motivation levels for quitting smoking than the general population, which is likely due to the recruitment strategy attracting those who were more motivated to participate in a tobacco cessation study. Because we only used insurance status to determine socioeconomically disadvantaged, some participants may not align with all characteristics that may describe socioeconomically disadvantaged.

### Conclusions and Future Work

Our project is the first to rigorously test the use of a recommender system for smoking cessation among individuals from socioeconomically disadvantaged backgrounds who smoke. In this paper, we have reported on protocols for conducting a rigorous trial. In addition to our primary analyses for the 3 aims, we will conduct exploratory analyses to assess the heterogeneity of treatment effects across these subgroups, which could yield valuable insights for future tobacco cessation efforts. Our follow-up papers will report on the results of our trial following the analysis plans described in this paper. Future studies will also explore implementing texting interventions in various clinical and public health settings.

## Appendix

Multimedia Appendix 1 Telephone screening script, consent form, baseline survey, and 6-month follow-up survey.

Multimedia Appendix 2 Example motivational messages (expert and peer).

Multimedia Appendix 3 Participant demographic and screening survey responses (N=757).

Multimedia Appendix 4 SPIRIT-AI checklist.

## Abbreviations

BPMF: Bayesian probabilistic matrix factorization
CO: carbon monoxide
IRB: institutional review board
NRT: nicotine replacement therapy
PCS: Perceived Competence Scale
REDCap: Research Electronic Data Capture
SDT: self-determination theory
SEM: structural equation modeling
UMass Chan: University of Massachusetts Chan Medical School
