# Correction: Testing a Machine Learning–Based Adaptive Motivational System for Socioeconomically Disadvantaged Smokers (Adapt2Quit): Protocol for a Randomized Controlled Trial

**DOI:** 10.2196/79873

**Published:** 2025-12-09

**Authors:** Ariana Kamberi, Benjamin Weitz, Julie Flahive, Kavitha Balakrishnan, Julianna Eve, Reem Najjar, Tara Liaghat, Daniel Ford, Peter Lindenauer, Sharina Person, Thomas K Houston, Megan E Gauvey-Kern, Jackie Lobien, Rajani S Sadasivam

**Affiliations:** 1 Division of Health Informatics and Implementation Science Department of Population and Quantitative Health Sciences UMass Chan Medical School Worcester, MA United States; 2 Division of Biostatistics and Health Services Research Department of Population and Quantitative Health Sciences UMass Chan Medical School Worcester, MA United States; 3 Department of Healthcare Delivery and Population Sciences University of Massachusetts Chan Medical School-Baystate Springfield, MA United States; 4 Institute for Clinical and Translational Research School of Medicine Johns Hopkins University Baltimore, MD United States; 5 Department of Internal Medicine Wake Forest University Winston-Salem, NC United States

In “Testing a Machine Learning–Based Adaptive Motivational System for Socioeconomically Disadvantaged Smokers (Adapt2Quit): Protocol for a Randomized Controlled Trial,” [1] the authors made one addition:

Kavitha Balakrishnan, BS

was added as an author to the study protocol. Neither the ‘Acknowledgments’ nor ‘Conflicts of Interest’ needed to be amended.

The correction will appear in the online version of the paper on the JMIR Publications website, together with the publication of this correction notice. Because this was made after submission to PubMed, PubMed Central, and other full-text repositories, the corrected article has also been resubmitted to those repositories.

## References

[1] Kamberi Ariana, Weitz Benjamin, Flahive Julie, Eve Julianna, Najjar Reem, Liaghat Tara, Ford Daniel, Lindenauer Peter, Person Sharina, Houston Thomas K, Gauvey-Kern Megan E, Lobien Jackie, Sadasivam Rajani S (2025). Testing a Machine Learning-Based Adaptive Motivational System for Socioeconomically Disadvantaged Smokers (Adapt2Quit): Protocol for a Randomized Controlled Trial. JMIR Res Protoc.

