# Supplementary material for: Testing a Machine Learning–Based Adaptive Motivational System for Socioeconomically Disadvantaged Smokers (Adapt2Quit): Protocol for a Randomized Controlled Trial
Source: JMIR Res Protoc. 2025 Apr 16;14:e63693. doi: 10.2196/63693 (PMC12044314; doi:10.2196/63693)
Supplement: Multimedia Appendix 1 [file resprot_v14i1e63693_app1.pdf]

TELEPHONE SCREENING SCRIPT
Adapt2Quit Study

Hi, my name is [*Name*] and I'm calling from [*Study Site*] to speak with [*Participant's Name*]. Is s/he available?

[If the person who answers the phone is not the participant: Ask if the participant is available or when they will be; call back later.]

[If no one answers: Do NOT leave a voicemail and hang up the phone at the 1st call. Leave a message on the 2nd call and say:

> *Hello, this message is for [Participant's name]. My name is [Name] and I am calling from [Study Site] to talk to you about a tobacco study called "Adapt2Quit." We sent you a letter about two weeks ago that describes the study. If you are interested in participating or would like to hear more about our study, please give me a call at [Number]. Thank you and have a nice day!*

[If the person who answers the phone is the participant:]

I'm calling today to see if you may be interested in participating in a voluntary research study called "Adapt2Quit." This study tests a computer program that selects and sends text messages for smokers. Do you have few minutes to talk now and learn more about this study?

[If "no time":]
When would be a good day and time to call you back to review the study? *[add callback time in REDCap and to study calendar]*

[If "not interested":]
Are you willing to share your reason for not participating ______________? Thank you for speaking with me today. I appreciate your time!

[If participant says they are not a current smoker:]

> That's good news, thank you for sharing! When did you quit? Can I ask you a few questions about your smoking history?

[If "yes":]

> Wonderful, thank you! I'll explain a bit more about the study now. If you chose to join the study, we will send you text messages over a 6-month period. The text messages are designed to support you to think about quitting smoking, even if right now you are not thinking about quitting. We may ask you to rate the messages based on if the message might help you quit smoking, either now or in the future. I'm going to ask you a few questions now to see if you are eligible for the study!

My first question is:

1. Have you smoked at least 100 cigarettes in your entire life? [Note: 5 packs = 100 cigarettes]

   ☐ Yes
   ☐ No

   [If no:]

I'm sorry, you are not eligible to participate in this study. Thank you for speaking with me today. I appreciate your time.

2. Do you now smoke cigarettes every day, some days, or not at all?
- ☐ Everyday
- ☐ Some days
- ☐ Not at all

[*If "Not at all"*:]

2b. Do you currently smoke cigarettes (smoked even 1 puff in the last 30 days)?
- ☐ No
- ☐ Yes

[If "no":]
I'm sorry, you are not eligible to participate in this study. Thank you for speaking with me today. I appreciate your time.

3a. Do you now smoke e-cigarettes every day?
- ☐ Everyday
- ☐ Some days
- ☐ Not at all

3b. Do you **only** smoke e-cigarettes?

- ☐ Yes
- ☐ No

[If "Yes" to Q#3b:]
I'm sorry, you are not eligible to participate in this study. Thank you for speaking with me today. I appreciate your time.

[If eligible for the study:]

Thank you; you are eligible to participate in this study. Does this study sound like something you would like to take part in?

[If "no"]: Are you willing to share your reason for not participating _______________? Thank you for speaking with me today. I appreciate your time!

[If yes]: Great! I will now review the consent form with you. Could I have your email address?

UNIVERSITY OF MASSACHUSETTS CHAN MEDICAL SCHOOL
COMMITTEE FOR THE PROTECTION OF HUMAN SUBJECTS IN RESEARCH

CONSENT TO PARTICIPATE IN A RESEARCH STUDY

Title: Adapt2Quit – A Machine-Learning, Adaptive Motivational System: RCT for smokers (Adapt2Quit)

Sponsor: National Institutes of Health

Investigator: Rajani Sadasivam, PhD

368 Plantation Street

Worcester, MA 01605

Daytime Phone Number: (508) 856-8924

Consent Version: Number 6.0

KEY INFORMATION

You are being invited to participate in a research study because you currently smoke cigarettes, are over 18 years of age, English speaking and able to read English at least at the second-grade level, and willing to receive and send study-related text messages.

Taking part in this research is voluntary and completely up to you. You are free to say no or to leave the research at any time. There will be no penalties or changes in the quality of the health care you receive, and you will not lose any benefits to which you are otherwise entitled.

The main question this study is trying to answer – We are testing whether the texting program we developed will help those who smoke to quit smoking. This texting program will ask your feedback on every text to select the best message for you as well as connect you to the Quitline (1-800-QUIT NOW). We will compare this with a program that sends you text messages to connect you to the Quitline (1-800-QUIT NOW), which can help people who smoke to quit. We would randomly assign (like flipping a coin) which program you will receive.

If you join this research, you will be asked to participate in some or all of the following activities:

- Attend an initial phone or Zoom call with the study team to determine your eligibility for the study.
- If eligible, complete the online consent process and a baseline survey by phone or Zoom call. If you do not have internet access and are unable to complete the online consent, we will mail a paper consent form to your home address and review the consent by phone once the form has been reviewed and received. This session will take about 30 minutes of your time.

UMass Chan Medical School IRB Approved

- You may receive text messages over a 6-month period. You may also receive a text asking you to rate these messages.
- You will receive a bi-weekly quitline text over a 6-month period. The text will ask whether you want to be connected to the quitline to help you quit smoking. If you reply 'yes,' our team will then refer you to the quitline and the quitline will contact you to setup the counseling sessions. This referral will include us sharing your name, phone number, and year of birth with the quitline.
- You will receive monthly texts over a 6-month period asking, "About how many cigarettes did you smoke in the past 7 days?"
- Responses to each text should take less than 1 minute.
- At the end of six months of participation, we will ask you to complete a telephone or Zoom call follow-up interview to assess your smoking status. This will take about 30 minutes of your time.
- At the end of six months of participation, we may ask you to complete a qualitative interview over the phone or Zoom to learn more about your experiences during the study. This interview will take about 30-60 minutes of your time. With your permission, this interview will be audio recorded using Zoom or digital recorder, transcribed, and then entered into the regulated environment through a secure Research Electronic Data Capture (REDCap) form.
- You may be asked to to complete carbon monoxide breath testing in-person or mailed to you if you are unable to come on-site.

You may not want to be in this study if you are uncomfortable with:

- Use of technology including text messaging
- Communicating with our staff on a phone or video call
- Sending and receiving study-related text messages

Risks: The risks of the study are not high. There may be minor inconveniences due to receiving the text messages at inconvenient times. There is a risk of accident or injury if you read or respond to text messages while driving, walking or doing other activities that require your attention. Another risk might be the accidental leak of your personal information; however, every precaution will be taken to prevent this.

Benefits: You may benefit from receiving the text messages, and/or being connected with the quitline, both of which may help you to reduce smoking or quit. Your alternative is to not take part in the research.

If you think you might like to participate in this research, please continue reading to learn more about the details of this study.

STUDY DETAILS

How many people will take part in this research?

UMass Chan Medical School IRB Approved

We expect to recruit about 750 individuals who smoke to participate in the study from three sites in the United States. University of Massachusetts Chan Medical School is one of the sites; the other two sites are Baystate Health in Springfield and Johns Hopkins in Maryland. We expect to enroll 100 participants at the UMass Chan site.

How long will I be in this research?

Your participation in the study won't last longer than 6-7 months including the time from the initial screening to the final follow-up.

What happens if I say yes, I want to be in this research?

If you say yes, we will ask you to participate in certain procedures (please see above **If you join this research** for the set of procedures). Participation in the study is voluntary and you are free to change your mind and discontinue participation in the study at any time.

A description of this clinical trial will be available on http://www.ClinicalTrials.gov with the trial number NCT04720625, as required by U.S. Law. This Web site will not include information that can identify you. At most, the Web site will include a summary of the results. You can search this Web site at any time.

Will you be collecting any specimens from me?

No specimens will be collected.

Could being in this research hurt me?

There may be minor inconveniences due to receiving the text messages at inconvenient times. There is a risk of accident or injury if you read or respond to text messages while driving, walking or doing other activities that require your attention. You do not have to respond any questions that you are not comfortable answering, and you can stop responding at any time.

Will it cost me any money to take part in this research?

This study will not cost you any money. You will use your text messaging and data plan to receive text messages.

Will I be given any money or other compensation for being in this study?

You will receive a $25 gift card for participating in the initial phone call, and $25 for completing the follow-up interview. If chosen to complete a qualitative interview, you may also receive an additional $50. If chosen to complete the carbon monoxide testing, we will also provide $50 for completing this activity whether in-person or remotely.

What happens if I am injured because I took part in this research?

If you are injured while in the study, seek treatment and contact the study coordinator as soon as you are able. The University of Massachusetts Chan Medical School does not provide funds for the treatment of research-related injury. If you are injured as a result of your participation in this study, treatment will be provided. You or your insurance carrier will be expected to pay the costs of this treatment. No additional financial compensation for injury or lost wages is available.

UMass Chan Medical School IRB Approved

You do not give up any of your legal rights by signing this form.

What are my responsibilities if I take part in this research?

If you take part in this research, you will be responsible for:

- Following the directions of the study team.
- Calling the research team at (508) 856-8924 if you have any questions.
- Calling the research team if you no longer wish to participate in the study.
- Not reading text messages while driving or walking or doing other activities that require your attention. Please make sure you are always choosing a safe time to read and respond to your texts. Please do not read or respond while driving.

What happens if I say yes, but I change my mind later?

You are free to leave the study at any time. This is called withdrawing. Your decision will not result in any penalty or loss of benefits. Data that we have already used will stay in the study database and cannot be removed in order to maintain the integrity of the research. However, you can ask us to destroy any information that identifies you.

Can I be removed from the research without my approval?

The person in charge of the research study or the sponsor can take you out of the study even if you do not want to leave. This may happen if the study is stopped by the funder or the University of Massachusetts Chan IRB.

We will tell you about any new information that may affect your health, welfare, or choice to stay in this research.

How will my information be stored and when will it be destroyed?

All trial data collected in the project will be stored in a regulated environment in the UMass Chan Medical School data center and all paper documents will be kept under a lock and key. Recorders will be stored in a locked drawer and zoom recordings will be downloaded in a secured file location. The data will be protected in the password-protected PI's UMass Chan Medical PQHS department folder. Data regarding the usage of the Quitline (for example, the number and dates of calls, whether nicotine replacement therapy (NRT) was requested, etc.) will be downloaded once for all study participants at the end of the study. Once collected, this information will also be stored in the regulated environment. The regulated environment provides applications a secure network for collecting and storing confidential data. The regulated environment has been securely configured to allow application access via the secure socket layer (HTTPS) protocol. The regulated environment is secured using hardware and software firewalls, along with access restrictions to provide the needed security protocols for the regulatory and Federal standards required. Access is restricted through a Virtual Private Network, a secure RSA token, and only restricted personnel are allowed access to the regulated environment.

UMass Chan Medical School IRB Approved

There is no limit on the length of time we will store your data. We will destroy the master list of identifiers after 6 years from the end of study recruitment.

It is possible that we might use the research data in other future research. We may also share data with researchers and companies that are not part of UMass Chan. In these cases, we will not share your name or other information that identifies you directly, and we will not come back to you to ask you for your consent.

Who has access to my information?

Signing this document means you allow us, the researchers in this study, and others working with us to use some protected health information for this research study.

Your health information and research records will be shared with the study team and with individuals and organizations that conduct or watch over this research, to conduct the study and to make sure it is conducted as described in this form. Information and records may be shared with:

- The research sponsor (the National Institutes of Health).
- People who work with the research sponsor.
- Federal and state government agencies, such as NIH and state auditors.
- The University of Massachusetts Chan Medical School and UMass Memorial Health Care, including the Institutional Review Board (IRB) and research, billing, and compliance offices.

We will protect your identifiable information from disclosure to others to the extent required by law, but we cannot promise complete secrecy.

We may publish the results of this research. However, we will keep your name and other identifying information confidential.

Any disclosure carries the potential for re-disclosure. Once your protected health information is disclosed, it may no longer be protected by federal privacy laws.

You may not be allowed to review some of the research-related information in your medical record until after the study is completed. When the study is over, you will have the right to access the information again.

Your authorization does not have an expiration date. If you change your mind, you have the right to revoke your authorization in writing or using the contact information at the beginning of this form. In such a case, you will not be allowed to continue to participate in the study. We will not collect any new information and may only use the information already collected for this research study. Your information may still be used and disclosed if you have an adverse event.

UMass Chan Medical School IRB Approved

You do not have sign this authorization. If you choose not to sign, it will not affect your treatment, payment, or enrollment in any health plans, or affect your eligibility for benefits. You will not be allowed to participate in the research study. Because the National Institutes of Health (NIH) funds this research, this study has a Certificate of Confidentiality. The Certificate keeps us from sharing your identifiable sensitive information collected for the research unless you allow us to do so. It also keeps us from being forced to release information that may identify you, as part of a court, legislative, administrative, or other proceeding.

There are times when the Certificate cannot be used. For example, we cannot refuse to give information to government agencies that oversee or fund research, such as the NIH or Food and Drug Administration (FDA). The Certificate also does not stop us from giving information to local government agencies, law enforcement personnel, or others if we suspect you or someone else is in danger or if we are required to do so by law.

The Certificate does not stop you from giving out information about yourself or your participation in the research. If you give an insurer, employer, or someone else your permission for us to release information, we will do so.

Will you share any results with me?

We are not planning to share data with our participants. If you would like us to try to reach you and share the data, please let us know. We will ask for your contact information.

Who can I talk to?

If you have questions, concerns, or complaints, or think this research has hurt you or made you sick, talk to the research team at the phone number listed on the first page.

This research is being overseen by an Institutional Review Board. An IRB is a group of people who perform independent review of research studies. You may talk to them at (508) 856-4261 or irb@umassmed.edu for any of the following:

- Your questions, concerns, or complaints are not being answered by the research team.
- You cannot reach the research team.
- You want to talk to someone besides the research team.
- You have questions about your rights as a research participant.
- You want to get information or provide input about this research.

Statement of Voluntary Consent

UMass Chan Medical School IRB Approved

I have read this form or have had it read to me. I have been told what to expect if I take part in this study, including possible risks and benefits, and have had the chance to ask questions. I know who to contact with future questions or concerns regarding this study. By consenting, I am volunteering to be in the "Adapt2Quit – A Machine-Learning, Adaptive Motivational System: RCT for smokers" research study.

☐ Yes

☐ No

Mail-in Participant ID Number: ______________

UMass Chan Medical School IRB Approved

STUDY_ID_________________________

UMass Chan
MEDICAL SCHOOL

Adapt2Quit

Research Study

Baseline Survey

We encourage you to answer all questions. Please follow instructions where noted.
Thank you for your participation!

STUDY_ID______________________

DEMOGRAPHICS

1. What is your age: _______________

2. Are you:
   - ☐ Male
   - ☐ Female
   - ☐ Non-binary
   - ☐ Prefer not to answer
   - ☐ None of the above: _________

3. Which of the following best describes your racial background? (select one or more of the following)
   - ☐ Black
   - ☐ White
   - ☐ Asian
   - ☐ Native American or Alaska Native
   - ☐ Native Hawaiian or Other Pacific Islander
   - ☐ Don't Know/Not Sure
   - ☐ Refused
   - ☐ Other

4. Do you consider yourself to be Hispanic or Latino, that is a person of Mexican, Puerto Rican, Cuban, South or Central American, or other Spanish culture or origin regardless of race?
   - ☐ Not Hispanic or Latino
   - ☐ Hispanic or Latino
   - ☐ Don't Know/Not Sure
   - ☐ Refused

5. What is the HIGHEST GRADE OR YEAR OF SCHOOL you completed?
   - ☐ Never attended school or only attended kindergarten
   - ☐ Elementary to grade 11 (some high school)
   - ☐ Grade 12 or GED (high school graduate)
   - ☐ College – 1 year to 3 years (some college)
   - ☐ College – 4 years or more (college graduate)
   - ☐ Don't Know/Not Sure
   - ☐ Refused

6. Are you?
   - ☐ Married
   - ☐ Divorced
   - ☐ Widowed
   - ☐ Separated
   - ☐ Never Married
   - ☐ A member of an unmarried couple

7. How would you describe your own health?
   - ☐ Excellent
   - ☐ Very Good
   - ☐ Good
   - ☐ Fair
   - ☐ Poor

8. How difficult is it for you to meet the monthly payments on your bills?
   - ☐ Extremely difficult
   - ☐ Very difficult
   - ☐ Somewhat difficult
   - ☐ Slightly difficult
   - ☐ Not difficult at all

9. How confident are you in filling out medical forms by yourself?
   - ☐ Not at all
   - ☐ A little bit
   - ☐ Somewhat
   - ☐ Quite a bit
   - ☐ Extremely

MEDICAL HISTORY

10. In the last 6 months, did you have to stay overnight in the hospital for any reason?
    - ☐ No
    - ☐ Yes

11. In general, would you say your **mental** health is:
    - ☐ Excellent
    - ☐ Very Good
    - ☐ Good
    - ☐ Fair
    - ☐ Poor

12. In the last 6 months, have you seen any health care provider for an emotional or mental health problem?
    - ☐ No
    - ☐ Yes

-_

SMOKING STATUS AND SOCIAL SUPPORT

13. What is your current smoking status?
    - ☐ I am not thinking about quitting
    - ☐ I am thinking about quitting
    - ☐ I have set a quit date
    - ☐ I quit today
    - ☐ I have already quit

14. How many cigarettes do you smoke per day?________________

15. How much money **per week** do you currently spend on tobacco products?
    - ☐ $0-25
    - ☐ $26-50
    - ☐ $51-75
    - ☐ $76-100
    - ☐ $100+

16. How soon after you wake up do you smoke your first cigarette (Fagerstrom Test for Nicotine)?
    - ☐ Within 5 minutes
    - ☐ 6-30 minutes
    - ☐ 31-60 minutes
    - ☐ After 60 minutes

17. During the past 12 months, have you stopped smoking for one day or longer because you were trying to quit smoking?
    - ☐ No
    - ☐ Yes

18. Have you ever visited a smoking cessation website?
    - ☐ No
    - ☐ Yes

19. Do you want to stop smoking cigarettes?
    - ☐ No
    - ☐ Yes

SMOKING ENVIRONMENT

20. Besides yourself, does anyone who lives in your home currently smoke cigarettes?
    - ☐ No
    - ☐ Yes

21. Is this person trying to quit?
    - ☐ N/A
    - ☐ No
    - ☐ Yes

22. Thinking about the people you know who smoke cigarettes, please list the number of smokers for each group below in the "# of smokers" column on the right:

**Group**	**# of smokers**
Immediate family members:	
Close friends, with whom you feel at ease to discuss private matters with:	

Other tobacco - e-cigarette use attitudes and beliefs

23. Have you ever tried an e-cigarette or electronic cigarette, a cigarette-looking electronic device that delivers nicotine vapor , even just one time?
    - ☐ No
    - ☐ Yes

24. How many days have you used an e-cigarette in the past 7 days?
- ☐ Every day
- ☐ Some days
- ☐ Not at all

25. Why did you use an e-cigarette?
- ☐ To quit smoking
- ☐ To cut down on my smoking
- ☐ To use in places where I was not allowed to smoke cigarettes
- ☐ Other
  If other, please elaborate: _________________

26. Besides cigarettes and e-cigarettes, do you smoke any other form of tobacco?
- ☐ No
- ☐ Yes (specify): _________________________

27. Do you currently use marijuana or cannabis (often called “weed” or “pot”):
- ☐ Every day
- ☐ Some days
- ☐ Not at all

-_

NRT use

28. Have you/Are you currently using any of the following *(check all that apply)*:
- ☐ Nicotine Patches (transdermal nicotine system)
- ☐ Nicotine Nasal Spray
- ☐ Nicotine Inhaler
- ☐ Nicotine Lozenge
- ☐ Chantix
- ☐ Nicotine Gum (Nicotine Polacrilex)
- ☐ Wellbutrin
- ☐ N/A

Quitline – 1-800-QUIT NOW

A quitline is a free national and state-wide tobacco cessation service that you can contact through a toll-free telephone number. Quitline staff are trained to help you deal with challenges and achieve your smoking goals. The first call takes about 20-30 minutes for registration, intake, and coaching. After that, calls are about 15-20 minutes on average, scheduled about 7-10 days apart. The standard program is a 5-call program. Quitline coaches are available 24/7 and can provide additional support by email, text, or chat. You may also be eligible to receive free nicotine replacement therapy, such as nicotine gum, lozenges, or patches.

29. Have you ever called a telephone quitline to help you quit smoking?
- ☐ No
- ☐ Yes

30. How likely would you be to call a smoking cessation telephone quitline in the future, for any reason?
    - ☐ Very likely
    - ☐ Somewhat likely
    - ☐ Somewhat unlikely
    - ☐ Very unlikely
    - ☐ Don't know

Perceived competence scale (PCS)

31. Please indicate the extent to which each statement is true for you, assuming that you were intending either to permanently quit smoking now or to remain permanently abstinent from smoking. Use the following scale:

Statements	Not at all true 1	2	3	Somewhat true 4	5	6	Very true 7
a. I feel confident in my ability to not smoke.	1	2	3	4	5	6	7
b. I now feel capable of not smoking.	1	2	3	4	5	6	7
c. I am able to not smoke anymore.	1	2	3	4	5	6	7
d. I am able to meet the challenge of not smoking.	1	2	3	4	5	6	7

Perceived Stress Scale

32. In the last month, how often have you felt that you were unable to control the important things in your life?
    - ☐ Never
    - ☐ Almost Never
    - ☐ Sometimes
    - ☐ Fairly often
    - ☐ Very often

33. In the last month, how often have you felt confident about your ability to handle your personal problems?
    - ☐ Never
    - ☐ Almost Never
    - ☐ Sometimes
    - ☐ Fairly often
    - ☐ Very often

34. In the last month, how often have you felt that things were going your way?
    - ☐ Never
    - ☐ Almost Never
    - ☐ Sometimes
    - ☐ Fairly often
    - ☐ Very often

35. In the last month, how often have you felt difficulties were piling up so high that you could not overcome them?
- ☐ Never
- ☐ Almost Never
- ☐ Sometimes
- ☐ Fairly often
- ☐ Very often

STUDY_ID________________________

Adapt2Quit

Research Study

6-Month Follow-Up Survey

We encourage you to answer all questions. Please follow instructions where noted.
Thank you for your participation!

STUDY_ID______________________

Date: _______/________/____________

SMOKING STATUS

1. Do you currently smoke cigarettes (smoked even 1 puff in the last 7 days)?
   - ☐ No [go to 1a, then skip to #4]
   - ☐ Yes

   1a. [if NO] How long has it been since you last smoked? ____________________

2. How soon after you wake up do you smoke your first cigarette (Fagerstrom Test for Nicotine)?
   - ☐ Within 5 minutes
   - ☐ 6-30 minutes
   - ☐ 31-60 minutes
   - ☐ After 60 minutes
   - ☐ Other

3. Currently, about how many cigarettes did you smoke per day?________________

4. About how many times in the last 6 months have you made what you would consider a "serious" attempt to quit smoking?

   If never quit, circle "0" 0 _______times

5. How many days have you used an e-cigarette in the past 7 days?
   - ☐ Every day
   - ☐ Some days
   - ☐ Not at all

6. Why did you use an e-cigarette?
   - ☐ To quit smoking
   - ☐ To cut down on my smoking
   - ☐ To use in places where I was not allowed to smoke cigarettes
   - ☐ Other

     If other, please elaborate: _________________

7. Besides cigarettes and e-cigarettes, do you smoke any other form of tobacco?
   - ☐ No
   - ☐ Yes (specify): _________________________

NRT use

8. In the last 6 months, have you used/are you currently using any of the following *(check all that apply)*:
   - ☐ Nicotine Patches (transdermal nicotine system)
   - ☐ Nicotine Nasal Spray
   - ☐ Nicotine Inhaler
   - ☐ Nicotine Lozenge
   - ☐ Nicotine Gum (Nicotine Polacrilex)
   - ☐ Chantix
   - ☐ Wellbutrin
   - ☐ N/A

Quitline

9. During the past 6 months, did you call a telephone quitline to help you quit smoking?
   - ☐ No
   - ☐ Yes

Perceived competence scale (PCS)

10. Please indicate the extent to which each statement is true for you, assuming that you were intending either to permanently quit smoking now or to remain permanently abstinent from smoking. Use the following scale:

**Statements**	**Not at all true 1**	**2**	**3**	**Somewhat true 4**	**5**	**6**	**Very true 7**
a. I feel confident in my ability to not smoke.	1	2	3	4	5	6	7
b. I now feel capable of not smoking.	1	2	3	4	5	6	7
c. I am able to not smoke anymore.	1	2	3	4	5	6	7
d. I am able to meet the challenge of not smoking.	1	2	3	4	5	6	7

11. We would like to know how much the past few months has influenced you. As a result of your experience, <u>what is your current smoking status?</u>
    - ☐ I am not thinking about quitting
    - ☐ I am thinking about quitting
    - ☐ I have set a quit date
    - ☐ I quit today
    - ☐ I have already quit

12. In the last 6 months, did you use any of the following strategies to help meet your smoking goals?

	Check all that apply
1. I talked with my doctor	☐
2. I asked for support from those around me to quit smoking	☐
3. I set a quit date	☐
4. I used Nicotine Replacement Therapy like the patch or gum	☐
5. I was given a prescription by my doctor to help quit smoking, such as Chantix (Varenicline) or Wellbutrin (Bupropion)	☐
6. I made a list of reasons to quit smoking to help me	☐
7. I used behavioral strategies like distraction or substitution	☐
8. I used counseling (I talked with a counselor, participated in group counseling)	☐
9. I called a quitline	☐
10. I vaped (used electronic cigarettes) instead of smoking cigarettes	☐
11. I joined a smoking cessation web program	☐
12. I used a smoking cessation mobile app	☐

As part of the Adapt2Quit study, we sent you text messages over the last 6 months and you rated them. We then selected the next message based on the messages you liked. Thinking about your overall experience with the text messaging system, please answer the following questions:

13. How much do you agree that the motivational text messages were helpful in meeting your smoking goals?
- ☐ Strongly Agree
- ☐ Agree
- ☐ Neutral
- ☐ Disagree
- ☐ Strongly Disagree

14. On a scale from 1-10, where 1 is 'not relatable at all' and 10 is 'extremely relatable,' how relatable/relevant were the text messages?

1	2	3	4	5	6	7	8	9	10
Not relatable at all				Neutral				Extremely relatable	

15. We asked you if you would like to be referred to the quitline. Was the quitline text message easy to understand? [see sample screenshot below]

    ☐ Yes
    ☐ No

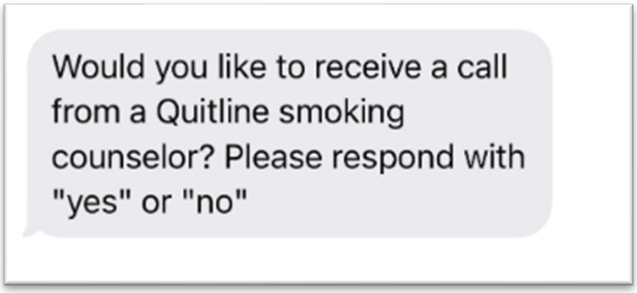

16. Thinking about the messages you received during the study, what were the things you liked about the messages? (optional)

    ____

17. Thinking about the messages you received during the study, what additional things would you have liked to see in the messages? (optional)

    ____

18. Was there anything that was not good for your health or privacy related to your participation in this study? (optional)

    ____

19. How much do you agree that rating the text messages was easy? [see sample screenshot below]

    ☐ Strongly Agree
    ☐ Agree
    ☐ Neutral
    ☐ Disagree
    ☐ Strongly Disagree

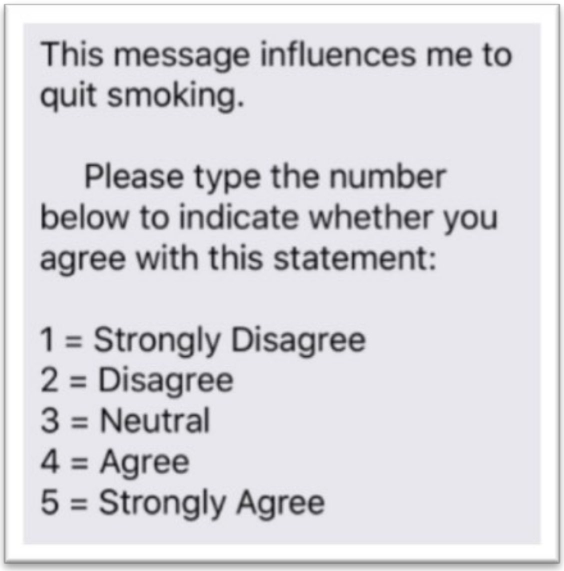

20. Did you understand that the messages changed because you rated them?

    ☐ Yes
    ☐ No

21. I would recommend the Adapt2Quit text message system to my friends and family.
   - ☐ Strongly Agree
   - ☐ Agree
   - ☐ Neutral
   - ☐ Disagree
   - ☐ Strongly Disagree

22. Would you be willing to be called for additional follow-up for this study or other research studies?
   - ☐ Yes
   - ☐ No

STUDY_ID_________________________

Adapt2Quit

Research Study

6-Month Follow-Up Survey

We encourage you to answer all questions. Please follow instructions where noted.
Thank you for your participation!

STUDY_ID________________________

Date: _______/________/____________

SMOKING STATUS

1. Do you currently smoke cigarettes (smoked even 1 puff in the last 7 days)?
   - ☐ No [go to 1a, then skip to #4]
   - ☐ Yes

   1a. [if NO] How long has it been since you last smoked? ____________________

2. How soon after you wake up do you smoke your first cigarette (Fagerstrom Test for Nicotine)?
   - ☐ Within 5 minutes
   - ☐ 6-30 minutes
   - ☐ 31-60 minutes
   - ☐ After 60 minutes
   - ☐ Other

3. Currently, about how many cigarettes did you smoke per day?________________

4. About how many times in the last 6 months have you made what you would consider a "serious" attempt to quit smoking?

   If never quit, circle "0" 0 _______times

5. How many days have you used an e-cigarette in the past 7 days?
   - ☐ Every day
   - ☐ Some days
   - ☐ Not at all

6. Why did you use an e-cigarette?
   - ☐ To quit smoking
   - ☐ To cut down on my smoking
   - ☐ To use in places where I was not allowed to smoke cigarettes
   - ☐ Other

     If other, please elaborate: _________________

7. Besides cigarettes and e-cigarettes, do you smoke any other form of tobacco?
   - ☐ No
   - ☐ Yes (specify): _________________________

NRT use

8. In the last 6 months, have you used/are you currently using any of the following *(check all that apply)*:
    - ☐ Nicotine Patches (transdermal nicotine system)
    - ☐ Nicotine Nasal Spray
    - ☐ Nicotine Inhaler
    - ☐ Nicotine Lozenge
    - ☐ Nicotine Gum (Nicotine Polacrilex)
    - ☐ Chantix
    - ☐ Wellbutrin
    - ☐ N/A

Quitline

9. During the past 6 months, did you call a telephone quitline to help you quit smoking?
    - ☐ No
    - ☐ Yes

Perceived competence scale (PCS)

10. Please indicate the extent to which each statement is true for you, assuming that you were intending either to permanently quit smoking now or to remain permanently abstinent from smoking. Use the following scale:

**Statements**	**Not at all true 1**	**2**	**3**	**Somewhat true 4**	**5**	**6**	**Very true 7**
a. I feel confident in my ability to not smoke.	1	2	3	4	5	6	7
b. I now feel capable of not smoking.	1	2	3	4	5	6	7
c. I am able to not smoke anymore.	1	2	3	4	5	6	7
d. I am able to meet the challenge of not smoking.	1	2	3	4	5	6	7

11. We would like to know how much the past few months has influenced you. As a result of your experience, <u>what is your current smoking status?</u>
    - ☐ I am not thinking about quitting
    - ☐ I am thinking about quitting
    - ☐ I have set a quit date
    - ☐ I quit today
    - ☐ I have already quit

12. In the last 6 months, did you use any of the following strategies to help meet your smoking goals?

	Check all that apply
1. I talked with my doctor	☐
2. I asked for support from those around me to quit smoking	☐
3. I set a quit date	☐
4. I used Nicotine Replacement Therapy like the patch or gum	☐
5. I was given a prescription by my doctor to help quit smoking, such as Chantix (Varenicline) or Wellbutrin (Bupropion)	☐
6. I made a list of reasons to quit smoking to help me	☐
7. I used behavioral strategies like distraction or substitution	☐
8. I used counseling (I talked with a counselor, participated in group counseling)	☐
9. I called a quitline	☐
10. I vaped (used electronic cigarettes) instead of smoking cigarettes	☐
11. I joined a smoking cessation web program	☐
12. I used a smoking cessation mobile app	☐

As part of the Adapt2Quit study, we sent you text messages over the last 6 months. Thinking about your overall experience with the text messaging system, please answer the following questions:

13. We asked you if you would like to be referred to the quitline. Was the quitline text message easy to understand? [see sample screenshot below]

- ☐ Yes
- ☐ No

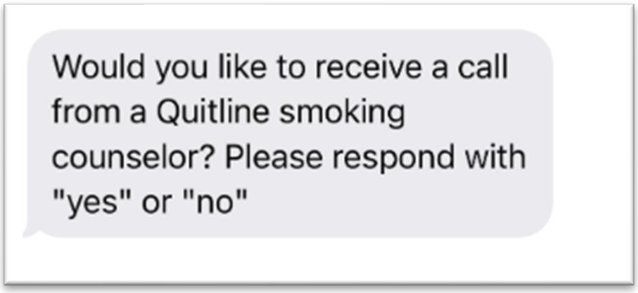

14. Was there anything that was not good for your health or privacy related to your participation in this study? (optional)

____

15. I would recommend the Adapt2Quit text message system to my friends and family.
- ☐ Strongly Agree
- ☐ Agree
- ☐ Neutral
- ☐ Disagree
- ☐ Strongly Disagree

16. Would you be willing to be called for additional follow-up for this study or other research studies?
- ☐ Yes
- ☐ No

A2Q Qualitative Interview Guide

PAT ID#__________________ Date___________________

Welcome back and thank you for helping us with our study!

Before we start, we want to make sure that you are aware that we are audio recording this session and will transcribe your feedback after it is over. Please do not mention your full name or anyone else's once the interview recording starts. We will also be writing down notes during the session.

Now that you have received and rated text messages for 6 months, we would like to ask you some questions about your experience during the study. Overall, how was your experience as a participant in the study?

*[**Note for the interviewer**: if a participant answers 'yes' or 'no' to a question, follow up with, "Can you tell me more about that?"]*

**Objective – To Investigate:**	**Questions**
1. Text message feedback	1.1 We would like to ask you about the motivational text messages we sent to you over the last 6 months. Can you please tell me your general thoughts about these text messages?<br><br>- Which messages were most memorable or motivating or stood out to you? Please tell us more about these.<br>- How could we improve the messages? Please tell us more.<br>- Were there certain topics that seemed to be missing from the messages? Please tell us more.<br>- Did you ever save a message to look back at it? Tell us more about why you did or didn't need to do this.<br>- Did you ever share the messages with someone else? Tell us more about why you did or didn't need to do this.<br>- How often and when would you have liked to receive the messages? Should we have sent more or less?<br><br>1.2 In these messages, we provided some tips and strategies to help you with reducing or quitting smoking. Can you tell us how you used the tips provided in the messages? Can you tell us more about how it was helpful or why you didn't use them?<br><br>- Did these messages help you to cut down the number of cigarettes you smoked or quit smoking?<br>- Did you recognize that some of our messages included tips/strategies written by other smokers? For example, 'Paul, a

	former smoker, suggested X.' Tell us more about what you thought about these messages.<br>- Do you suggest any specific strategies to help smokers with reducing or quitting smoking?<br><br>1.3 In our study, we asked you to rate the messages. For example, we asked you to tell us if you strongly agreed, agreed, neutral, disagreed, or strongly disagreed with a message. Tell us about your experience with rating the messages.<br><br>- Was it easy to reply with just a number to rate the messages?<br>- It looks like you rated X messages. What helped you to rate the messages? Tell us more about why you didn't rate the other messages.<br>- What was difficult or were there challenges with rating the messages?<br>- Did you understand that the messages became more personalized because you rated them? Tell us more about how you felt your ratings made messages more helpful to you.<br>- Tell us why you didn't think the messages became more personalized, and how could we have made it clearer to you?
2. Quitline use and feedback	2.1 In the study, we sent you messages asking, 'Would you like to receive a call from a quitline counselor?' Tell us what you thought about this message.<br><br>- Our records show that <u>you asked to be referred</u>. Tell us more of what you thought about the steps to connect to the quitline. Was it easy to respond to this message? What did you expect to happen? What actually happened? Did you talk with the quitline? Why? Why not?<br>(I) What motivated you to connect with the Quitline?<br>- Our records show that <u>you did not ask</u> to be connected to the qutiline. Was there a reason you did not ask to connect?<br>(I) When you got the Quitline message, was it clear that you would be referred? Or were these messages 'lost in the crowd' with the motivational messages?<br>(II) What might have encouraged you to be referred to the Quitline?
3. Recruitment/Follow-up procedures	3.1 Could you please describe what you liked or disliked when we initially reached out asking you to join our study?<br><br>- Was it easy to sign up for and be part of the study? Was anything difficult?

	3.2 What were your experiences with the research team once you decided to participate in the study?<br><br>- Did you feel that the research team gave you enough information about the study activities?
4. Coping/support for quitting	4.1 Did you seek help with quitting during the time you were participating in the study? Tell us more about the support you used.<br><br>- Did you have family support? Friends who helped?<br>- What other things did you try? *(prompts: use of NRT, distraction, substitutions, talked with family, doctors, etc.)*<br>- What other support systems would have been helpful to you if available/offered?
5. Technology use and attitudes	5.1 Did you use any other technology, like smart phone apps or websites, to help you help you reduce smoking/quit?<br><br>- If yes, which ones? What did you like about them? What was helpful or could have been improved?<br>- If no, why not? What may have motivated you to use them?<br><br>5.2 Can you tell us your overall thoughts about using technology for health/quitting tobacco?<br><br>- Have you/any members of your family used technology for health reasons before?<br>- What types of technology have you used?<br>- What do you think about making health interventions more like a game (adding points, leaderboards, badges, virtual rewards, connecting to real prizes/rewards, progress reports)? Would that be helpful?
6. E-cigarette use	6.1 Did you use e-cigarettes (vape) at all in the last 6 months? Tell us more about the use of e-cigarettes.<br><br>- Why did you use them? Did you think it would help with quitting smoking?<br>- Which product did you use? Did it contain nicotine?<br>- Did you smoke cigarettes and vape at the same time? Why?
7. Overall impression of the study/future improvements	7.1 We are interested in ways to make the study better for future participants. Is there anything we didn't already cover that you think could improve the study?<br><br>- What did we do well?

	- In future studies, what other technologies or functions would you like to see included?<br>- Would you recommend this study to a friend?
