# Supplementary material for: Testing a Machine Learning–Based Adaptive Motivational System for Socioeconomically Disadvantaged Smokers (Adapt2Quit): Protocol for a Randomized Controlled Trial
Source: JMIR Res Protoc. 2025 Apr 16;14:e63693. doi: 10.2196/63693 (PMC12044314; doi:10.2196/63693)
Supplement: Multimedia Appendix 2 [file resprot_v14i1e63693_app2.docx]

| **Type** | **Multimedia Appendix 2: Example Motivational Messages (Expert and Peer)** | **Content Coding** |
| --- | --- | --- |
| **Peer 1** | Darcy, a 39-year-old, has thought about the financial savings of quitting smoking and says: Plan to save all the money you spend on cigarettes just like you were still buying them and put it away to see how much you can save and use it for something great :) | ***Socio-Cultural Attributes-Money***    ***Behavioral strategies*** |
| **Peer 2** | Kelly, a 28-year-old, has described the role family and friends should play in quitting smoking as…Being patient and encouraging. They should use positive reinforcement and tell you on a regular basis that you are doing a good job. Don't let your efforts go unnoticed | ***Socio-Cultural Family/Friend*** |
| **Expert 1** | Even though smoking really harms your body, it's not too late – you can undo much of the damage by quitting smoking now. | ***Health: Physical Functioning -*** |
| **Expert 2** | Long term risks of smoking include heart attacks and stroke, cancer of all types, osteoporosis, long-term disability, and the need for extended care. Think about what your life will be like if you keep smoking. | ***Health-Medical***  ***problems*** |
