# Supplementary material for: Testing a Machine Learning–Based Adaptive Motivational System for Socioeconomically Disadvantaged Smokers (Adapt2Quit): Protocol for a Randomized Controlled Trial
Source: JMIR Res Protoc. 2025 Apr 16;14:e63693. doi: 10.2196/63693 (PMC12044314; doi:10.2196/63693)
Supplement: Multimedia Appendix 3 [file resprot_v14i1e63693_app3.docx]

**Multimedia Appendix 3: Table S1** Participant demographic and screening survey responses (N=757).

| Characteristics | | | Values, n (%) |
| --- | --- | --- | --- |
| **Gender** | | | |
|  | Female | | 486 (64.2) |
|  | Male | | 269 (35.5) |
|  | Trans man | | 1 (0.1) |
|  | None of the above | | 1 (0.1) |
| **Race (not mutually exclusive)** | | | |
|  | American Indian or Alaskan native | | 19 (2.5) |
|  | Asian | | 8 (1.1) |
|  | Black or African American | | 265 (35) |
|  | Native Hawaiian or Other Pacific Islander | | 2 (0.26) |
|  | White | | 387 (51.1) |
| **Ethnicity** | | | |
|  | Hispanic or Latino | | 121 (16) |
|  | Not Hispanic or Latino | | 634 (83.8) |
| **Highest grade or year of school completed** | | | |
|  | Elementary to grade 11 (some high school) | | 113 (14.9) |
|  | Grade 12 or GED^a^ (high school graduate) | | 285 (37.6) |
|  | College—1 to 3 years (some college) | | 261 (34.4) |
|  | College—≥4 years (college graduate) | | 98 (12.9) |
| **Marital status** | | | |
|  | Married | | 145 (19.2) |
|  | Divorced | | 148 (19.6) |
|  | Widowed | | 52 (6.9) |
|  | Separated | | 37 (4.9) |
|  | Never married | | 274 (36.1) |
|  | A member of an unmarried couple | | 47 (6.2) |
| **Health** | | | |
|  | Excellent | | 28 (3.7) |
|  | Very good | | 113 (14.9) |
|  | Good | | 270 (35.7) |
|  | Fair | | 261 (34.4) |
|  | Poor | | 85 (11.2) |
| **Difficulty paying bills monthly** | | | |
|  | Extremely difficult | | 71 (9.4) |
|  | Very difficult | | 69 (9.1) |
|  | Somewhat difficult | | 229 (30.2) |
|  | Slightly difficult | | 156 (20.6) |
|  | Not difficult at all | | 231 (30.5) |
| **Are you confident in filling out medical forms by yourself?** | | | |
|  | Not at all | | 37 (4.9) |
|  | A little bit | | 47 (6.2) |
|  | Somewhat | | 101 (13.3) |
|  | Quite a bit | | 123 (16.2) |
|  | Extremely | | 449 (59.3) |
| **Currently spend on tobacco products per week** | | | |
|  | US $0-$25 | | 165 (21.8) |
|  | US $26-$50 | | 242 (32) |
|  | US $51-$75 | | 145 (19.2) |
|  | US $76-$100 | | 123 (16.2) |
|  | US >$100 | | 70 (9.2) |
| In the last 6 months, did you stay overnight in the hospital? | | | 123 (16.2) |
| **General mental health** | | | |
|  | Excellent | | 102 (13) |
|  | Very good | | 163 (22) |
|  | Good | | 250 (33) |
|  | Fair | | 195 (26) |
|  | Poor | | 46 (6.1) |
| In the last 6 months, did you see a health care provider for an emotional or a mental health problem? | | | 284 (38) |
| **How soon after you wake up do you smoke your first cigarette?** | | | |
|  | Within 5 minutes | | 299 (39.4) |
|  | 6-30 minutes | | 230 (30.4) |
|  | 31-60 minutes | | 114 (15.1) |
|  | After 60 minutes | | 109 (14.4) |
| **Want to stop smoking cigarettes?** | | | |
|  | No | | 25 (3.3) |
|  | Yes | | 725 (95.8) |
|  | I do not smoke now | | 5 (0.7) |
| In the past 12 months, stopped smoking for >1 day | | | 379 (50.1) |
| Ever visited a smoking cessation website | | | 97 (12.8) |
| **Besides yourself, does someone who lives in your home currently smoke cigarettes?** | | | 291 (38.4) |
|  | That person is trying to quit | | 120 (15.8) |
| Ever tried an e-cigarette | | | 494 (65.2) |
| **Number of days an e-cigarette was used in the past 7 days** | | | |
|  | Everyday | | 38 (5) |
|  | Some days | | 70 (9.2) |
|  | Not at all | | 508 (67.1) |
| **Why did you use an e-cigarette?** | | | |
|  | To quit smoking | | 155 (20.5) |
|  | To cut down on my smoking | | 148 (19.6) |
|  | To use in places where I was not allowed to smoke cigarettes | | 58 (7.7) |
|  | Not at all | | 98 (12.9) |
|  | Other | | 118 (15.6) |
| **Currently use marijuana or cannabis** | | | |
|  | Every day | | 147 (19.4) |
|  | Some days | | 144 (19) |
|  | Not at all | | 461 (60.9) |
| **Have you or are you currently using any of the following (not mutually exclusive)?** | | | |
|  | Nicotine patches (transdermal nicotine system) | | 380 (50.2) |
|  | Nicotine nasal spray | | 6 (0.8) |
|  | Nicotine inhaler | | 16 (2.1) |
|  | Nicotine lozenge | | 130 (17.2) |
|  | Chantix | | 202 (26.7) |
|  | Nicotine gum (nicotine polacrilex) | | 267 (35.3) |
|  | Wellbutrin | | 155 (20.5) |
| Ever called a telephone Quitline to help you quit smoking | | | 126 (16.6) |
| **Likely to call a smoking cessation telephone Quitline in the future** | | | |
|  | Very likely | | 150 (19.8) |
|  | Somewhat likely | | 242 (32) |
|  | Somewhat unlikely | | 82 (10.8) |
|  | Very unlikely | | 150 (19.8) |
|  | Do not know | | 132 (17.4) |
| **I feel confident in my ability to not smoke** | | | |
|  | Not at all true | | 154 (20.3) |
|  | 2 | | 31 (4.1) |
|  | 3 | | 59 (7.8) |
|  | Somewhat true | | 256 (33.8) |
|  | 5 | | 74 (9.8) |
|  | 6 | | 46 (6.1) |
|  | Very true | | 137 (18.1) |
| **I now feel capable of not smoking** | | | |
|  | Not at all true | | 205 (27.1) |
|  | 2 | | 54 (7.1) |
|  | 3 | | 53 (7) |
|  | Somewhat true | | 224 (29.6) |
|  | 5 | | 80 (10.6) |
|  | 6 | | 47 (6.2) |
|  | Very true | | 93 (12.3) |
| **I am able to not smoke anymore** | | | |
|  | Not at all true | | 310 (41) |
|  | 2 | | 46 (6.1) |
|  | 3 | | 53 (7) |
|  | Somewhat true | | 168 (22.2) |
|  | 5 | | 61 (8.1) |
|  | 6 | | 37 (4.9) |
|  | Very true | | 79 (10.4) |
| **I am able to meet the challenge of not smoking** | | | |
|  | Not at all true | | 92 (12.2) |
|  | 2 | | 37 (4.9) |
|  | 3 | | 42 (5.6) |
|  | Somewhat true | | 215 (28.4) |
|  | 5 | | 94 (12.4) |
|  | 6 | | 73 (9.6) |
|  | Very true | | 201 (26.6) |
| **In the last month, how often have you felt that you were unable to control the important things in your life?** | | | |
|  | Never | | 199 (26.2) |
|  | Almost never | | 120 (15.8) |
|  | Sometimes | | 256 (33.8) |
|  | Fairly often | | 83 (11) |
|  | Very often | | 93 (12.3) |
| **In the last month, how often have you felt confident about your ability to handle your problems?** | | | |
|  | Never | | 195 (25.8) |
|  | Almost never | | 148 (19.6) |
|  | Sometimes | | 246 (32.5) |
|  | Fairly often | | 86 (11.4) |
|  | Very often | | 77 (10.2) |
| **In the last month, how often have you felt that things were going your way?** | | | |
|  | Never | | 52 (6.9) |
|  | Almost never | | 82 (10.8) |
|  | Sometimes | | 316 (41.7) |
|  | Fairly often | | 166 (22) |
|  | Very often | | 139 (18.4) |
| **In the last month, how often have you felt difficulties were piling up so high that you could not overcome them?** | | | |
|  | Never | | 195 (25.8) |
|  | Almost never | | 148 (19.6) |
|  | Sometimes | | 246 (32.5) |
|  | Fairly often | | 86 (11.4) |
|  | Very often | | 77 (10.2) |
| **Number of smokers in** | | | Median (IQR) |
|  | | Immediate family members | 2  (0-4) |
|  | | Close friends | 2  (1-5) |
| Number of cigarettes smoked per day | | | 10  (7-20) |

^a^GED: General Educational Development.
